# New Applications for Submucosal Tunneling in Third Space Endoscopy

**DOI:** 10.1097/MCG.0000000000001762

**Published:** 2022-09-09

**Authors:** Huifang Xia, Yan Peng, Xiaowei Tang

**Affiliations:** Department of Gastroenterology, Affiliated Hospital of Southwest Medical University Luzhou, Sichuan Province, China

***To the Editor:***

We read with great interest the article by Karanfilian and Kahaleh,^1^ in which third space endoscopy (TSE) can be used to treat gastrointestinal motility disorders and neoplasms throughout the digestive tract. This review mentioned the main TSE procedures and their applications, including peroral endoscopic myotomy, gastric peroral endoscopic myotomy, Zenker peroral endoscopic myotomy, submucosal tunneling for endoscopic resection, endoscopic submucosal tunnel dissection, peroral endoscopic tunneling for restoration of the esophagus, and per-rectal endoscopic myotomy.^1^

Although we agree the usages and efficacy for these offshoosts of TSE, we also found that some potential indications of TSE were not discussed in the review. First, Chavan et al^2^ reported that endoscopic resection of a complex gastric duplication cyst using a submucosal tunneling technique successfully. Second, a study demonstrated that submucosal tunneling for endoscopic resection is an effective method for en bloc resection of predominant extraluminal growing subepithelial tumors or extragastrointestinal tumors.^3^ Eight patients were enrolled in this study, the average procedure time was 67±4.4 minutes. The rates of curative en bloc resection and en bloc retrieval was 100% and 87.5%, respectively, with a mean length of stay of 3 days and no major adverse events or deaths. After a mean follow-up period of 10.0±2.1 months, there were no residual tumors on surveillance images.^4^ Third, gastric peroral endoscopic myotomy was also reported to used for treat infantile hypertrophic pyloric stenosis, which is a common disease requiring laparoscopic or open pyloric myotomy in infants. A study reported a 35-day-old infant underwent this procedure with a good recovery and no postoperative complications.^5^ Last, submucosal tunneling endoscopic biopsy and myotomy is also an effective approach to diagnose unknown esophageal stenosis.^6^

Based on the current evidence, TSE has quickly developed since it was firstly applied in clinical practice in 2010, and it may still be a rapidly growing field of therapeutic endoscopy. With the advancement of devises and equipment, TSE will be used to treat more diseases in the future.
